# Implications for Extracellular Matrix Interactions With Human Lung Basal Stem Cells in Lung Development, Disease, and Airway Modeling

**DOI:** 10.3389/fphar.2021.645858

**Published:** 2021-05-12

**Authors:** Shana M. Busch, Zareeb Lorenzana, Amy L. Ryan

**Affiliations:** ^1^ Hastings Center for Pulmonary Research, Division of Pulmonary, Critical Care and Sleep Medicine, Department of Medicine, University of Southern California, Los Angeles, CA, United States; ^2^ Department of Stem Cell Biology and Regenerative Medicine, University of Southern California, Los Angeles, CA, United States

**Keywords:** airway modeling, chronic lung disease, cellular niche, extracellular matrix, basal stem cells, regeneration

## Abstract

The extracellular matrix (ECM) is not simply a quiescent scaffold. This three-dimensional network of extracellular macromolecules provides structural, mechanical, and biochemical support for the cells of the lung. Throughout life, the ECM forms a critical component of the pulmonary stem cell niche. Basal cells (BCs), the primary stem cells of the airways capable of differentiating to all luminal cell types, reside in close proximity to the basolateral ECM. Studying BC-ECM interactions is important for the development of therapies for chronic lung diseases in which ECM alterations are accompanied by an apparent loss of the lung’s regenerative capacity. The complexity and importance of the native ECM in the regulation of BCs is highlighted as we have yet to create an *in vitro* culture model that is capable of supporting the long-term expansion of multipotent BCs. The interactions between the pulmonary ECM and BCs are, therefore, a vital component for understanding the mechanisms regulating BC stemness during health and disease. If we are able to replicate these interactions in airway models, we could significantly improve our ability to maintain basal cell stemness *ex vivo* for use in *in vitro* models and with prospects for cellular therapies. Furthermore, successful, and sustained airway regeneration in an aged or diseased lung by small molecules, novel compounds or via cellular therapy will rely upon both manipulation of the airway stem cells and their immediate niche within the lung. This review will focus on the current understanding of how the pulmonary ECM regulates the basal stem cell function, how this relationship changes in chronic disease, and how replicating native conditions poses challenges for *ex vivo* cell culture.

## Introduction

The extracellular matrix (ECM) is a three-dimensional (3-D) network of extracellular macromolecules that provides structural, mechanical, and biochemical support for the cells and tissues that make up complex organisms. It is becoming increasingly apparent that the ECM plays a vital role in development and maintenance of healthy adult tissues. This review will focus on the ECM of the tracheo-bronchial airways, discussing our current knowledge of how the pulmonary ECM facilitates lung development, influences the stem cell niche, changes with chronic disease, and poses challenges for cell culture in an *ex vivo* environment. If we are to look forward to regenerative strategies for the human lung it is critical that we understand the mechanisms by which the immediate cellular environment of the basal airway stem cell regulates its long-term regenerative capacity for restoring functional mucociliary clearance.

The pulmonary ECM changes significantly throughout lung development. In addition to providing a progressive scaffold for the complex structure of the lung from the trachea to alveoli, the ECM influences stem cell specification and differentiation to the diverse cell types of the adult lung (McGowan, 1992; Burgstaller et al., 2017; Zhou et al., 2018). This is thought to occur through compartment-specific ECM composition, substrate-stiffness, and other mechanical properties, as well as biochemical signaling (Yayon et al., 1991; McGowan, 1992; Coraux et al., 2002; Izvolsky et al., 2003; Arteaga-Solis et al., 2012; Enomoto et al., 2013; Burgstaller et al., 2017; Zhou et al., 2018). Several growth factors and cytokines have been shown to influence lung development and regulate ECM deposition and basal cell (BC) phenotype. Fibroblast growth factor 10 (FGF10) and transforming growth factor beta 1 (TGF-β1) are both examples of such factors that interact with and/or influence the pulmonary ECM (Yayon et al., 1991; Sekine et al., 1999; Izvolsky et al., 2003; Alcorn et al., 2008; Arteaga-Solis et al., 2012; Li et al., 2017; Schittny, 2017; Velden et al., 2018). In the adult lung, the ECM forms a critical component of the pulmonary stem cell niche. Basal cells (BCs), the primary stem cells of the airways, reside in close proximity to the basolateral ECM and will be the primary focus of this review due to their interactions with the basement membrane and importance in regeneration of the mucociliary epithelium of the airways. While BCs are known to have the capacity to regenerate all the cells comprising the epithelium of the cartilaginous airways, the mechanisms regulating this are still poorly understood. There is increasing evidence that supports the existence of multiple sub-types of BCs *in vivo* with varying capacity for differentiation (Watson et al., 2015; Greaney et al., 2020; Travaglini et al., 2020; Carraro et al., 2020a; Carraro et al., 2020b) and distinct phenotypic changes in disease (Watson et al., 2015; Greaney et al., 2020; Travaglini et al., 2020; Carraro et al., 2020a; Carraro et al., 2020b). Despite an increase in our knowledge of both the pulmonary ECM and lung BCs there is still a paucity of information on how the ECM may influence BC stemness. As such, it is imperative that we develop a better understanding of the interactions between BCs and the ECM. Studying BC-ECM interactions is also important for the development of therapies for chronic lung diseases in which ECM alterations are accompanied by an apparent loss of the lung’s regenerative capacity (Gohy et al., 2015; Jonsdottir et al., 2015; Burgstaller et al., 2017; Ghosh et al., 2017). Such disorders include asthma. chronic obstructive pulmonary disease (COPD) and idiopathic pulmonary fibrosis (IPF) (Burgess et al., 2016; Burgstaller et al., 2017).

The impact of the ECM on cell survival and phenotype becomes apparent in an *ex vivo* culture environment. While a variety of culture matrices have been developed commercially, such as Matrigel® and PureCol®, to recreate the native ECM composition and mechanical properties, numerous studies have reported significant differences in phenotype and differentiation between native BCs and those expanded *ex vivo* (Zabner et al., 2003; Cortiella et al., 2010; Greaney et al., 2020; Carraro et al., 2020a; Carraro et al., 2020b). Furthermore, these matrices are poorly defined and thus variable batch to batch. Such discrepancies pose a great challenge for both healthy and diseased airway modeling, as we are unable to determine, for instance, whether a change in BC phenotype during *ex vivo* culture reflects the same BC attrition observed with aging or injury *in vivo* or is just an artifact of inadequate culture conditions. The complexity and importance of the native ECM in the regulation of BCs is highlighted as we have yet to create an *in vitro* culture model that is capable of the long-term expansion of BCs while maintaining their phenotypic stability (Zabner et al., 2003; Cortiella et al., 2010; Liu et al., 2012; Mou et al., 2016; Gentzsch et al., 2017; Peters-Hall et al., 2018; Greaney et al., 2020; Carraro et al., 2020b). To circumnavigate the difficulties of culturing airway cells on an artificial matrix, some have turned to decellularization and recellularization of the lung or trachea (Cortiella et al., 2010; Ott et al., 2010; Price et al., 2010; Badylak et al., 2011b; Au - Bonvillain et al., 2013; Keane et al., 2015; Uriarte et al., 2018; Greaney et al., 2020). Decellularization methods attempt to remove all cellular material, leaving behind the intact ECM (Ott et al., 2010; Price et al., 2010; Badylak et al., 2011b; Uriarte et al., 2018). With present techniques, some DNA and other cytoplasmic and/or nuclear material is usually retained by the scaffold, but the composition and mechanical properties of the ECM are largely preserved (Badylak et al., 2011b; Uriarte et al., 2018). Several laboratories have attempted to repopulate the ECM with *ex vivo* expanded cells (Cortiella et al., 2010; Au - Bonvillain et al., 2013; Gilpin et al., 2014; Keane et al., 2015; Burgstaller et al., 2018; Gilpin and Wagner, 2018; Uriarte et al., 2018; Greaney et al., 2020; Ravindra et al., 2021). While this has yielded promising results, there are many aspects, particularly in the recellularization process, that must be refined before decellularization/recellularization can be widely used in clinical applications.

Determining the precise nature of the interactions between the pulmonary ECM and BCs is, therefore, a vital component of BC regulation in lung development, regeneration, and disease pathogenesis. As lungs age, or acquire disease, their capacity for functional regeneration depletes; understanding how ECM-BC interactions are altered by aging or disease should inform new therapeutic approaches for restoring long-term airway function. In addition, improving *ex vivo* cell culture will create a more physiological model of disease offering a superior platform for evaluating the mechanisms that drive chronic lung disease and providing a pathway to breakthroughs in the development of regenerative therapeutics, including cellular therapy. In this review, the role of the ECM in lung development, disease, and as a niche for basal stem cells will be reviewed and complement recent reviews focusing more on the ECM in the distal-alveolar airspace (Tschumperlin, 2015; Gu et al., 2018; Zhou et al., 2018).

### Lung Development and the ECM

To understand the importance of the ECM in regulating BCs, it is essential to first discuss its role as a key component in regulating lung development and maintaining homeostasis (McGowan, 1992; Zhou et al., 2018). The composition of the pulmonary ECM changes with maturation from the fetal, to neonatal, and subsequently adult lung tissue (McGowan, 1992; Coraux et al., 2002; Burgstaller et al., 2017; Gilpin et al., 2017; Zhou et al., 2018). There are two principal concepts surrounding the role of ECM in lung development: 1) it provides a scaffold for cells and contributes to the mechanical properties of the lung, while supporting signaling between multiple cell types, and 2) its production and deposition is controlled by many cells throughout lung development (McGowan, 1992). In early lung morphogenesis, a respiratory diverticulum (or lung bud), is generated from the foregut endoderm in the human embryo approximately 5 weeks after conception (Warburton et al., 2010; Zhou et al., 2018). In humans, development then continues in four stages: pseudoglandular (5–17 weeks of gestation), canalicular (16–25 weeks), terminal saccular (24–32 weeks), and alveolar (32 or 36 weeks extending to childhood or early adolescence) (Burri, 1984; Warburton et al., 2010; Schittny, 2017; Zhou et al., 2018). Each stage is briefly discussed below, and we also refer the readers to comprehensive reviews on airway development (Burri, 1984; McGowan, 1992; Mund et al., 2008; Warburton et al., 2010; Schittny, 2017).

#### The Pseudoglandular Stage

The pseudoglandular stage is marked by airway branching. Branching morphogenesis is facilitated by ECM components including fibronectin, syndecan, laminin, and tenascin, along with interactions between the epithelium and mesenchyme (McGowan, 1992; Zhou et al., 2018). The cells primarily responsible for the production of such ECM proteins are pulmonary fibroblasts (Blaauboer et al., 2014; Kendall and Feghali-Bostwick, 2014; White, 2015). Epithelial branching can be induced by transplanting mesenchyme from an actively branching region into a normally unbranched region, demonstrating the importance of epithelial interactions with the mesenchyme (Shannon et al., 1998). The ECM expresses proteoglycans (PGs) decorin, lumican, and biglycan, along with collagens I, III, and VI at the epithelial-mesenchymal interface forming a sleeve around the bronchiolar ducts (Godoy-Guzman et al., 2012). Sulfated proteoglycans, heparin sulfates (HS), in the ECM may also contribute to the regulation airway branching due to their ability to regulate growth factor binding and signaling (Yayon et al., 1991; Aviezer et al., 1994; Izvolsky et al., 2003; Zhang et al., 2012). FGF-10 deficient mice do not survive post-natal due to lack of lung development, while the trachea is formed, pulmonary branching morphogenesis is disrupted (Yayon et al., 1991; Sekine et al., 1999; Izvolsky et al., 2003). HS are expressed in the lung mesenchyme at prospective bud sites in close proximity to FGF-10 expressing regions, and highly sulfated HS are also found in the basement membranes of epithelial tubule branches (Izvolsky et al., 2003; Zhang et al., 2012). This suggests that FGF-10′s ability to induce budding is significantly changed by the developmental patterns of HS sulfation (Izvolsky et al., 2003). By the conclusion of the pseudoglandular stage, all of the prospective conducting airways have been formed and the acinar outlines have appeared (Coraux et al., 2002; Warburton et al., 2010; Schittny, 2017).

#### The Canalicular and Saccular Stage

There are three critical events that occur during the canalicular stage: 1) the appearance of the distal alveolar air sac (acinus), 2) the development of the air-blood interface and differentiation of the pulmonary epithelium, and 3) the commencement of surfactant production (McGowan, 1992). Type 1 and 2 alveolar epithelial cells (AT1 and AT2 respectively), the latter being responsible for surfactant synthesis, arise from the differentiation of the glycogen-rich cuboidal epithelial cells of the future alveolar ducts (Schittny, 2017). AT1s, with AT2s integrated between them, form the thin lining of the alveolar ducts and sacculi (Schittny, 2017). The future air-blood barrier will form at the locations where the capillary endothelium comes into close contact with the AT1s (Schittny, 2017). By the end of the canalicular stage, the airways have been divided to form the future alveolar, also known as transitory, ducts (McGowan, 1992).

During the terminal saccular stage, air spaces continue to widen, and the air-blood interface becomes more closely related through the rearrangement of capillaries (McGowan, 1992; Warburton et al., 2010; Schittny, 2017). A significant decrease in the mass of the mesenchymal ECM accompanies this sophistication of the membrane between the capillary and alveolar wall (Burri, 1984; McGowan, 1992). This process seems to be mediated by elastin, as an organized deposition of elastin fibrils occurs beneath the transitory duct epithelium (McGowan, 1992; Schittny, 2017). Elastin, a structural ECM protein, is principally responsible for the elasticity of the alveolar walls and its deposition is maximal along the sites of future secondary crests (McGowan, 1992). To facilitate alveolar septation the precursor of elastin, tropoelastin, is produced and cross-linked by lysyl oxidase (Zhou et al., 2018). The elevation and suspension of the secondary crests that subdivide the primary saccules is a consequence of elastin deposition (Amy et al., 1977; Zhou et al., 2018). The importance of elastin for developing lung structure during the saccular stage is demonstrated when epithelial-driven inflammation is induced in murine models. Such inflammation disrupts elastin fiber organization and causes a decreased regulation in elastin assembly components such as fibulins 4/5, lysyl oxidase, and fribrillin-1 leading to impaired structural development during the saccular stage (Benjamin et al., 2016). Inflammation, as a result incomplete lung development, has also been implicated in human infants born prematurely (Kramer et al., 2009). The saccular stage appears to be an intermediate stage in which branching is concluded and the scaffold for future alveolarization is set into place.

#### The Alveolar Stage

In humans, alveolarization begins at approximately 20 weeks post-conception and is not completed until at least 7 years of age; in mice however, alveolarization takes place entirely postnatally (Mund et al., 2008; Warburton et al., 2010). The saccules are divided into alveoli by the formation of secondary crests that appear as narrow ridges (Amy et al., 1977; Branchfield et al., 2016). It has been demonstrated that secondary crests develop, through the deposition of elastic tissue, at the juxtaposition of a capillary, AT1 cell, and an epithelial cell (Amy et al., 1977). These observations suggest that portions of the primary saccular wall are tethered by the elastin and collagen fibers in the expanding secondary crest while the free regions of the wall enlarge outward into alveoli (Amy et al., 1977). FGF signaling is considered to be the director of elastin deposits during this stage by restricting the expression of the elastogenic machinery in the mesenchyme to allow for controlled and ordered formation of elastic ECM (Li et al., 2017). Alveolar myofibroblasts are considered to be the elastin producing cells of the lung due to their physical proximity to sites of elastin deposition (Vaccaro and Brody, 1978; Noguchi et al., 1989). In addition, platelet derived growth factor subunit A (PDGF-A) has been demonstrated to have an essential and highly specific function in lung development by promoting differentiation of fibroblasts to alveolar myofibroblasts and, as a consequence, alveolarization (Bostrom et al., 1996). PDGF-A deficient mice surviving birth show failed alveolar septation leading to lung emphysema (Bostrom et al., 1996; Lindahl et al., 1997). This appears to be the result of a lack of alveolar myofibroblasts and their associated elastin deposits (Bostrom et al., 1996). The degree of sulfation of proteoglycans (PGs) is also thought to play a role in alveolus formation. Sulfation of PGs is affected by sulfatases, activated by sulfatase-modifying factor 1 (SUMF1), through the removal of sulfate groups (Arteaga-Solis et al., 2012). Mice lacking the SUMF1 enzyme show normal lung development until the start of alveolarization when an increase in sulfated PGs is detected in the lung parenchyma along with a decrease in alveolar septa formation (Arteaga-Solis et al., 2012). These mice also showed an increase in TGF-β1 signaling (Arteaga-Solis et al., 2012). Removal of sulfatase activity seems to increase the deposition of sulfated PGs which leads to a deregulation of TGF-β1 signaling and a halt in alveolarization (Arteaga-Solis et al., 2012).

#### Adult Lung ECM Composition

Two major structural types compose the adult pulmonary ECM. First, basement membranes, thin glycoprotein sheets, line the basal surface of the epithelia and endothelia, and also envelop muscle, peripheral nerve cells, and fat (Sannes and Wang, 1997; Moore et al., 2005; Burgstaller et al., 2017). Second, interstitial matrices interconnect structural cells and maintain 3-D structure and biomechanical characteristics through a loose, fibril-like meshwork (Burgstaller et al., 2017). ECM proteins, glycosaminoglycans, and modifying enzymes assemble into insoluble composite materials to provide a binding interface for the hundreds of secreted proteins within the lung (Burgstaller et al., 2017; He et al., 2017; Wigen et al., 2019). This allows for the complex signaling that is thought to direct cell function and differentiation. Collagen and elastin are the two major ECM proteins of the lung. While collagens (types I, II, III, V, and XI) provide overall structure to the lung, elastic fibers are responsible for elastic recoil (Burgstaller et al., 2017; Mereness et al., 2018). Glycoproteins, fibulins, elastin microfibril interface-located proteins, and elastin-crosslinking lysyl oxidases are other proteins that are associated with elastic fibers (Ge et al., 2015; Liu et al., 2016; Burgstaller et al., 2017; Philp et al., 2018; Ito et al., 2019). PGs contribute to the viscoelasticity of the lung due to their hydrophilic nature (Burgstaller et al., 2017; Iravani et al., 2020). Post-translational modifications of ECM proteins, such as enzymatic or chemical crosslinking, glycation and glycosylation, and oxidation, allow for further structural and/or function diversity (Burgstaller et al., 2017; Merl-Pham et al., 2019; Rani et al., 2021). The remainder of this review will focus on current knowledge of how the ECM regulates lung stem cell function, and we refer readers to Burgstaller and colleagues (Burgstaller et al., 2017) for an in-depth review of the adult pulmonary ECM composition.

### The ECM and Regulation of Stem Cells

Decoding the regenerative capacity of the lungs is a vital step toward identifying potential therapies for chronic lung diseases. This has proven to be difficult because the lung is relatively quiescent under normal conditions with infrequent divisions of progenitor cells to maintain the respiratory epithelium (Beers and Morrisey, 2011; Stabler and Morrisey, 2017). As such, it is challenging to study epithelial regeneration under steady-state conditions, especially in relevant human cells, and artificial injury to activate stem cells and regenerate airways is required in animal models (Zhu et al., 2007; Rock et al., 2009; Mereness et al., 2018; Yuan et al., 2019). Additionally, there are limited studies on how the ECM facilitates and regulates stem cell differentiation and maintenance, and the relationship between changes in ECM composition in diseased states and the impact on the stem cell niche has yet to be fully elucidated. As BCs are known to be the major stem cell population in the conducting airways, we will focus on their interactions with the ECM *in vivo* and in *ex vivo* models.

#### The Pulmonary Stem Cell Niche

BCs are multipotent stem cells that reside in close proximity to the basal lamina in the pseudostratified epithelium of the tracheobronchial tree (Rock et al., 2009; Donne et al., 2015). They are present in approximately equal proportions to ciliated and secretory cells in the conducting airways (Rock et al., 2009; Donne et al., 2015). With their ability to self-renew and differentiate into all luminal cell types (Hong et al., 2004; Rock et al., 2009), BCs are thought to be important in maintaining epithelium homeostasis. As previously mentioned, the lung has a relatively slow cell-turnover rate during homeostasis (Beers and Morrisey, 2011; Stabler and Morrisey, 2017). To date BCs have been characterized by their expression of transcription factor tumor protein 63 (TP63), cytokeratin 5 (KRT5), podoplanin (PDPN), nerve growth factor receptor (NGFR), and cytokeratin 14 (KRT14) (Rock et al., 2009; Donne et al., 2015); however, the situation is likely more complex. New transcriptomic data has emerged that suggests the presence of a number of BC subpopulations (Watson et al., 2015; Smirnova et al., 2016; Carraro et al., 2020a). Each sub-type of BC may have a different physiological and functional role in airway maintenance and regeneration. A collaborative study by Carraro *et al.* has identified 5 BC sub-types in the native human proximal epithelium (Carraro et al., 2020a). The sub-types include one highly expressing canonical markers such as TP63, KRT5, and cytokeratin 15 (KRT15), and another with proliferative characteristics including DNA topoisomerase II alpha, and the marker of proliferation antigen Ki-67 (Carraro et al., 2020a). Of the various BC sub-populations, it has been suggested that ciliated and secretory “primed” cells exist as intermediates between BCs and their differentiated progeny (Watson et al., 2015; Carraro et al., 2020a). Watson *et al.* proposes that multipotent BCs give rise to luminal precursors (BLPs) that show upregulation of cytokeratin 8 (KRT8), and that these BLPs eventually differentiate to secretory or ciliated cells (Watson et al., 2015). Furthermore, the concept of “hillock” cells, originally described by Montoro and colleagues, was reconfirmed in a study that identified two populations within this KRT13 expressing “hillock” population, one which was notably high in markers of squamous/cornified epithelium (Montoro et al., 2018; Deprez et al., 2020). Further studies will be required to determine the precise functional differences that specifically define the BC sub-populations observed and determine if they are universally expressed in humans and other species.

Due to the important role of the ECM in directing lung development, it is thought to perform an equally important role in the maintenance of the pulmonary stem cell niche and the regulation of regeneration. These mechanisms and interactions between BCs and the ECM are historically poorly studied and defined; however, in more recent years there has been a greater push to advance our understanding in this area. Age related changes in the ECM have been shown to have a significant impact on epithelial phenotype and gene expression (Godin et al., 2016; Gilpin et al., 2017). Isolated human BCs demonstrated higher proliferative markers, PCNA and Ki67, and a higher percentage of living cells when cultured on a scaffold derived from a human early post-natal lung than an adult lung (Gilpin et al., 2017). The glycoproteins Fibrillin-2 and Tenascin-C were found to be significantly enriched in the post-natal lung and labeled as potential mediators of the reported results (Gilpin et al., 2017). Lung growth that occurs most robustly during the early post-natal period may explain why proliferation-stimulating ECM components are more highly expressed during this phase. In another study, collagen VI (COLVI) was found *in vivo* in a position to interact with epithelial cells in both the airways and alveoli (Mereness et al., 2018). In an *in vitro* experiment, primary human lung epithelial cells displayed a heightened rate of “healing” in response to scratch injury when plated on COLVI as opposed to Matrigel, collagen I (COLI), or a combination of Matrigel and COLI or COLVI (Mereness et al., 2018). Studies that follow up on these initial observations to determine the precise BC-ECM interactions that occur in early post-natal lung compared to the adult lung. This knowledge could inform on the development of better *in vitro* culture systems as well as identify new regulators of BC stemness and proliferation.

One of the early studies on the airway BC transcriptome identified gene clusters postulated to be associated with the cells ability to maintain the structural integrity of the airway epithelium (Hackett et al., 2011). Over 201 genes in the focal adhesion pathway overlapped with BCs inclusive of these associated with adherens junction formation, ECM-receptor interactions, and regulation of the actin cytoskeleton. Among these genes overrepresented in BCs were 5 laminin subunits (α, β1, β3, γ1, γ3), and 6 integrin subunits (α3, α5, α6, β1, β4, β6) associated with cell-cell and cell-ECM interactions (Evans et al., 2001; Hackett et al., 2011). To date most transcriptomic studies have included minimal validation of the findings with no specific functional characterization (Hackett et al., 2011; Hackett et al., 2012; Xu et al., 2016; Carraro et al., 2020a; Carraro et al., 2020b). The BC signature highlights the array of integrins, extracellular laminins and collagen, and adaptor proteins including actinin, vinculin and filamin essential for functional interaction with other cells and the ECM and also demonstrates differences between mouse and human BCs. For example, ITGA5 and ITGB6 are expressed in human, but not mouse BCs and, in humans, ITGA5 is known to mediate fibronectin-dependent epithelial cell proliferation through activation of EGFR (Kuwada and Li, 2000). Recently we have gained significant transcriptomic insight into BC subpopulations, however the field now needs to determine the regional-specific distribution and also validate the functional properties, cell: cell, and cell: ECM interactions that are relevant to homeostasis and to disease pathogenesis. Information acquired at the transcriptomic level does not always correlate to changes at the functional and physiological level (Kuwada and Li, 2000).

Understanding how the ECM influences the regeneration of an injured airway epithelium is a pre-requisite to the development of regenerative therapeutics for patients with acute and chronic lung disease. The scratch injury, mentioned above, is one model of airway wound repair in which epithelial-ECM interactions can be evaluated. Initial migration of airway BCs involves the formation of heterodimers of integrin α and β subunits, with the ECM expressing the ligands for integrins expressed on BCs regulating ECM-cell adhesion (Coraux et al., 2008; Hamilton et al., 2020). The importance of the various integrin subunits for BC migration directly depends on the ECM and, therefore, many *ex vivo* culture systems do not accurately reflect the specific process of *in vivo* airway regeneration. Matrix proteins, especially cellular fibronectin, are key in the successful migration of BCs and blocking their function substantially impairs wound repair (Hocking and Chang, 2003). It has been long established that as lungs age there is an age dependent change in the expression of fibronectin in the alveolar regions of the lung (D'Errico et al., 1989) and recent studies have supported such changes in the ECM, however there is still a paucity of information on how such age-related changes correlate to tissue formation and degradation and how ECM signals regulate injury repair and disease progression (Meiners et al., 2015; Brandenberger and Mühlfeld, 2017; Burgess et al., 2019). Given the increasing amount of data documenting ECM changes in the aging lung it is important that we start to understand the aging-related signature and its implications in the repair and regeneration of the lung epithelium.

In addition to being regulated by the matrix, BCs are also implicated in the production of ECM. During wound repair, the production of matrix components by BCs is influenced by the secretion of factors, such as TGF-β, either from BCs themselves or from inflammatory cells in the airways. The functional implications of these secreted factors remain somewhat unclear. For example, treatment with TGF-β impairs airway epithelial sheet migration due to increased cellular adhesion via integrins (Neurohr et al., 2006) and, conversely, TGF-β1 has also been shown to upregulate MMP2 accelerating the rate of epithelial repair (Coraux et al., 2008). Matrix metalloproteases (MMPs) regulate ECM secreted by the wound closing migratory BCs, these include MMP-9 (also known as gelatinase B), MMP-3 and MMP-11. These reparative BC often also express vimentin suggesting that acquisition of a mesenchymal phenotype is needed for effective wound closure via cell migration. Studies of mouse tracheal BCs have also demonstrated similar upregulation of markers of an epithelial to mesenchymal transition (EMT) (Velden et al., 2018). BCs subsequently plated on matrix secreted by TGF-β1-treated BCs have increased expression of EMT markers and acquire a spindle-like, fibroblastic appearance (Alcorn et al., 2008; Gohy et al., 2015; Velden et al., 2018; Adams et al., 2020).

BC-ECM interactions, although extensively investigated in the process of would repair, have been substantially less studied in the subsequent differentiation and restoration of functional mucociliary clearance. As BCs progress toward potential cellular therapy applications a detailed understanding of such interactions will be critical. Furthermore, determining the mechanisms driving changes in cellular phenotype and stemness during the *ex vivo* expansion of BCs will rely upon this information to design methods for effective cellular expansion while maintaining BC stemness. Many *ex vivo* culture systems study epithelial function and regeneration in monocultures comprising of epithelium alone. While these models can be used to study cell-matrix interactions critical modulation by exogenous influences such as inflammatory cells and their secreted mediators, and the surrounding mesenchyme limits the physiological interpretation of the model system. In the next section we will focus on how ECM components and mechanics are critical for maintaining BC stemness in an *in vitro* culture environment.

#### Culture and Airway Modeling

A variety of medias, matrices, and techniques are commonly used to culture BCs and/or drive differentiation into airway epithelium via culture at an air-liquid-interface (ALI) (Jiang et al., 2018) or in 3-D organoids or spheroids (Barkauskas et al., 2017). Commercially available ECMs used for BC expansion include Matrigel® (a protein mixture including laminin, collagen IV, and enactin that is derived from mouse tumor cells) (Hughes et al., 2010; Greaney et al., 2020) and PureCol® (a combination of collagens I and III) (Dodmane et al., 2018). A more extensive inventory of ECMs suitable for BC expansion and differentiation can be found in Table 1. However, BCs that are isolated and expanded *ex vivo* lose stemness within a few passages, differentiation capacity is reduced, and expression of genes that are critical for function, such as the cystic fibrosis transmembrane regulator (CFTR), decline (Zabner et al., 2003; Peters-Hall et al., 2018). In ALI cultures, primary BCs show a rapid linear decrease in CFTR function and ciliated cell formation with increased passages (Zabner et al., 2003; Peters-Hall et al., 2018). Often by passage 4, a significant decrease in differentiation capacity and CFTR expression/function is observed, and by passage 10 growth tends to stop (Zabner et al., 2003; Peters-Hall et al., 2018). In addition, airway epithelium produced by differentiation at ALI shows notable deviations from native samples. In the Carraro *et al.* study, 5 BC subtypes were identified in native human bronchial epithelium; however, only four subtypes were found in their ALI generated epithelia (Carraro et al., 2020a). While three subtypes were similar between the native and ALI samples, the fourth ALI subtype was high in KRT14 and lacked a native counterpart (Carraro et al., 2020a). The presence of a unique BC subtype in the ALI generated samples suggests that this subtype developed in response to culture conditions and demonstrates that the ALI culture platform may not be completely analogous *to in vivo* airway conditions.

**TABLE 1:** Matrices available for the culture of pulmonary basal stem cells.

Matrix (ECM)	Composition	Biological source	References
Matrigel®^a^ Geltrex®^a^ Cultrex®^b^	Laminin, collagen IV, enactin, heparin sulfate proteoglycans, growth factors	Murine engelbreth-holm-swarm tumor	Hughes et al. (2010); Badylak et al. (2011b)
PureCol®	97% Type I atelocollagen, 3% Type III collagen	Bovine hide	Dodmane et al. (2018)
Type I Collagen	Collagen I	Rat tail	Mereness et al. (2018)
Type IV Collagen	Collagen IV	Human cell culture	Gilpin et al. (2017)
Fibronectin	Fibronectin (Human plasma)	Human plasma	Hamilton et al. (2020)
Laminin	Laminin	Human placenta, human fibroblasts, murine engelbreth-holm-swarm tumor	Hamilton et al. (2020)

a Multiple varieties available including high concentration and reduced growth factor.

b Variety optimized for stem cell culture available.

Advanced media formulations have also extended our capacity to effectively maintain stem cell characteristics over prolonged *in vitro* expansion. The addition of a combination of three small molecules to the media, Rho-associated protein kinase (ROCK) inhibitor, dorsomorphin homolog 1 (DMH-1) [inhibitor of bone morphogenic proteins (BMP) signaling], and A-83-01 (inhibitor of TGF-β1 signaling), allows for extended airway BC expansion in the undifferentiated state; this is thought to be the result of dual SMAD inhibition (Mou et al., 2016). Cells grown in this media were positive for TP63 and KRT5, markers of multipotent epithelial BCs, and also expressed cell proliferation marker Ki-67, expanding for 18–25 passages without a loss of replicative potential (Mou et al., 2016). BMP and TGF-β1 inhibitors produced similar results when added to commercially available stem cell culture mediums bronchial epithelial cell growth medium (BEGM) and primary human airway epithelial cell media (HTEC) (Mou et al., 2016).

Prior to the addition of dual SMAD inhibitors to the media, the gold standard for preservation of stemness across serially passaged human bronchial epithelial cells (HBECs) was the addition of Rho kinase inhibitor (Y-27632) and co-culture on a layer of fibroblast feeder cells, specifically NIH-3T3-J2 cells (Liu et al., 2012; Gentzsch et al., 2017; Peters-Hall et al., 2018). Cells reprogrammed in this way are referred to as “conditionally reprogrammed cells” (CRCs). They retain a normal karyotype and remain non-tumorigenic (Liu et al., 2012). However, ALI cultures derived from CRC cells still show morphological differences and reduced CFTR expression compared to non-CRC ALIs (Gentzsch et al., 2017). Recently, a modified CRC (Mod-CRC) method has been presented that demonstrates improved preservation of differentiation capacity and CFTR function of HBECs (Peters-Hall et al., 2018). In a modified-CRC method, BEGM is used along with 2% O_2_, as opposed to a serum free expansion media and 21% O_2_ used in traditional CRC expansion (Peters-Hall et al., 2018).

To overcome culture constraints and improve the viability of BCs *ex vivo*, researchers are now exploring the decellularization of lung and airway tissues to more closely recapitulate the ECM and mechanical properties seen *in vivo* (Ott et al., 2010; Price et al., 2010; Wagner et al., 2014; Gilpin and Wagner, 2018)*.* Decellularization methods strive for total removal of the cellular material, while preserving the structure, protein and molecular composition, and mechanical properties of the ECM scaffold (Badylak et al., 2011b; Keane et al., 2015; Uriarte et al., 2018). The ultimate goal is to re-populate this scaffold with stem cells to completely regenerate a functional organ or tissue (Badylak et al., 2011b; Uriarte et al., 2018). While currently practiced methods of decellularization leave much of the ECM intact, including the structural geography of the airways, alveoli and vessels, there is a reduction in many components such as elastin, collagen, and sulfonated PGs (Price et al., 2010; Pouliot et al., 2016). Creation of a functional tissue from a decellularized scaffold has been attempted for multiple organs including heart, liver, urinary bladder, trachea, and lung (Cortiella et al., 2010; Badylak et al., 2011b). Decellularized lungs have been successfully re-populated with cells (Cortiella et al., 2010; Ott et al., 2010; Au - Bonvillain et al., 2013; Halabi and Mecham, 2018); however, keeping distinct cell populations in their proper areas and creating functional gas-exchange units has not been achieved (Uriarte et al., 2018). Ott and colleagues were successful in perfusing repopulated rat lung scaffolds with blood and performing ventilation, however transplantation of the lungs was only able to maintain gas exchange for 6 h (Ott et al., 2010). Although bio scaffolds generated from ECM of decellularized source tissues have been successfully used in the clinic (Badylak et al., 2011a; Dziki et al., 2016; Keane et al., 2017), for the trachea they have been unsuccessful due to the immediate need for a complete epithelium capable of functional mucociliary clearance. While an exciting advance to the field there is still a long way to go before the concept of a bioartificial lung will be a therapeutic reality.

**FIGURE 1:**
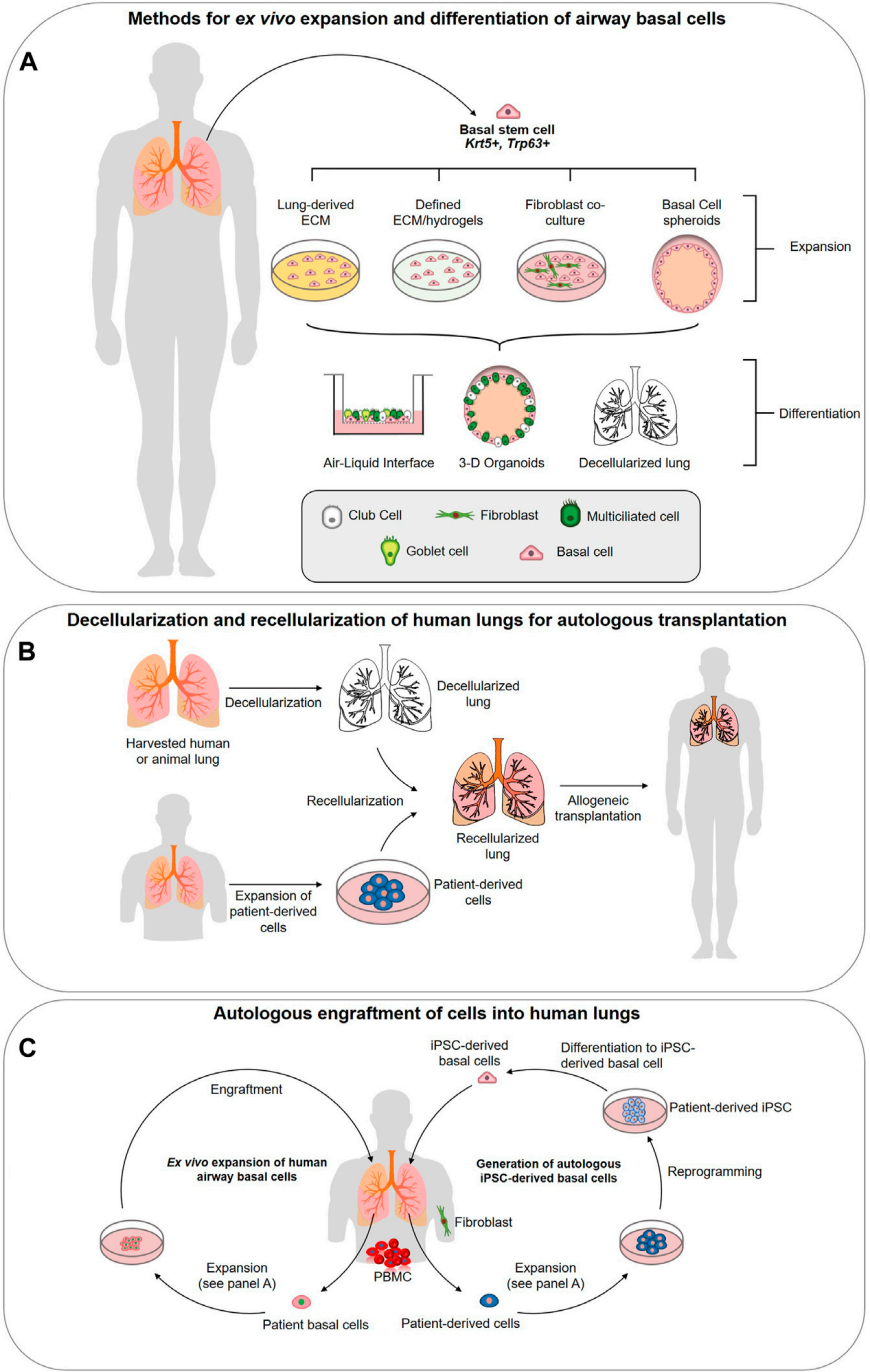
Strategies for utilizing airway basal stem cells in regenerative medicine approaches. **(A)** *Methods for ex vivo expansion and differentiation of airway basal cells*. Basal cells (BCs) can be isolated from the human lung epithelium and expanded in 3-D culture, co-culture with 3T3 fibroblasts, or potentially on specifically designed hydrogels or extracellular matrix (ECM)-derived from decellularized lungs. Expanded basal cells are capable of complete differentiation into a pseudostratified airway epithelium at the air-liquid interface, in 3-D spheroid cultures or in decellularized lung scaffolds. **(B)** *Decellularization and recellularization of human lungs for autologous transplantation.* Decellularization of human donor lungs, followed by recellularization with patient-derived cells (generated via methods described in panels c (primary airway cell expansion) and d (iPSC-derived basal cell generation. The concept is designed to achieve functional lung tissues or indeed, entire lungs, for autologous transplantation. **(C)** *Ex vivo expansion of human airway basal cells.* BCs can be isolated from human lung biopsy or bronchoscopy and expanded on defined ECM to maintain stemness and phenotype. The goal is to engraft these stem cells into the lung to restore airway function. *Generation of autologous iPSC-derived basal cells.* Using accessible cells such as peripheral blood mononucleocytes (PBMCs) or fibroblasts from skin biopsies, iPSC can be generated via reprogramming. Protocols now exist for their precise differentiation to lung basal cells (Hawkins et al., 2020) generating a potentially unlimited source of autologous cells for engraftment.

Although decellularization and recellularization techniques must be significantly refined before they are capable of generating a functional lung, researchers are demonstrating that there are advantages to using a native ECM scaffold for BC culture and epithelium generation (Figure 1A). Recently, a study by Greaney *et al.* compared the differences in differentiation of murine BCs across four different culture platforms: organoid, ALI, decellularized trachea, and decellularized lung (Greaney et al., 2020). They concluded that the decellularized or “engineered” scaffolds produced the most mature epithelial tissues *in vitro* over the artificial culture platforms (Greaney et al., 2020). Demonstrating the regional specificity of ECM and effect on differentiation, the engineered trachea contained the most mature proximal epithelium and ciliated cells, while the engineered lung showed early surfactant production suggesting distalization (Greaney et al., 2020). In the artificial platforms, the organoid culture presented a less developed epithelium and a non-physiological EMT population was observed at ALI (Greaney et al., 2020). This study supports the results of previous work by Cortiella *et al.* which compared the attachment, differentiation, and development of complex tissue of murine embryonic stem cells (ESCs) when expanded on several artificial culture matrices, including Matrigel and COLI, and whole decellularized lung (Cortiella et al., 2010). The lung scaffold produced more differentiated ESCs of epithelial and endothelial lineages, retained a higher ratio of living cells, and showed evidence of differentiated ESCs arranging into 3-D structures similar to those seen *in vivo* (2010). Such comprehensive studies have yet to be carried out for human airways and will be essential if a functional lung derived from a repopulated ECM scaffold is to become reality.

Instead of using a decellularized lung a scaffold for repopulation, some researchers are further processing native ECM into a hydrogel for 2-D or 3-D culture. These hydrogels exhibit characteristics such as self-assembly, architecture, and mechanical properties of the native tissue (Pouliot et al., 2016; de Hilster et al., 2020; Petrou et al., 2020). Mesenchymal stem cells, for example, encapsulated in hydrogels derived from porcine lung demonstrate attachment, proliferation, and viability at a comparable or greater level than cells grown in commercially available collagen-based matrices (Pouliot et al., 2016). Hydrogels have also been generated from human lungs that retained native stiffness in the range of 15–60 Pa (Pouliot et al., 2016; de Hilster et al., 2020). Changes in ECM-derived hydrogel stiffness induce significant changes in cultured cells. Petrou *et al.* reported that on hydrogels with increased elastic moduli, similar to native fibrotic ECM, ECM producing myofibroblasts showed heightened expression of myofibroblast transgenes and fibroblasts demonstrated a 60% increase in levels of collagen 1a1 and alpha smooth muscle actin compared to soft matrices (Petrou et al., 2020). These results may have significant implications for the understanding of cellular changes in response to fibrotic lung diseases. Further development of 2-D culture matrices derived from native pulmonary ECM will have important implications for 2-D culture of BCs and airway modeling, however, to date, there have been relatively few studies investigating the culture of airway BCs on these hydrogels. One recent study by Ravindra and colleagues compared HBEC attachment and functional differentiation on tracheal and urinary bladder ECM substrates. (Ravindra et al., 2021). Both hydrogels successfully supported cell proliferation and generation of a continuous basement membrane, however, while both substrates supported epithelial differentiation, this was not quantified or functionally evaluated. Critical next steps are to understand the components of the hydrogels that specifically support functional specification of the tracheal epithelium (Ravindra et al., 2021).

Most recently, progress in the development and use of synthetic ECM materials for cell culture have emerged. Biomaterial inks have been developed from regenerated silk fibroin (SF) and 2,2,6,6-tetramethylpiperidine-1-oxyl (TEMPO)-oxidized bacterial cellulose (OBC) nanofibrils and engineered for 3D printing of lung tissue scaffolds (Huang et al., 2020). Another group has developed a tissue-specific hybrid bioinks from the naturally occurring polymer alginate along with ECM derived from decellularized tissue (De Santis et al., 2021). In both models HBECs were able to adhere and proliferate, maintaining expression of basal cell markers TP63 and KRT5. De Santis and colleagues evaluated differentiation over a month on 3D bio-printed human airways composed of regionally specified airway smooth muscle and human bronchial epithelial cells and observed basolateral localization of BCs and evidence of differentiation to mucus-producing cells (MUC5AC^+^) and ciliated cells shown directly through scanning electron microscopy and alpha-tubulin expression (De Santis et al., 2021). The results of this study do demonstrate that a combination of exogenously and endogenously derived ECM materials supports epithelial development and offers the potential of printing customized airway scaffolds which has not yet been achieved with native ECM alone. Huang suggested that by manipulating the alignment of the OBC nanofibrils within the scaffold, ECM remodeling could be recreated and consequently manipulate cell fate (Huang et al., 2020). Unfortunately, neither study comprehensively evaluated functional differentiation, mucociliary clearance, and regenerative capacity, all core criteria in creating bio scaffolds for lung repair. (Huang et al., 2020) (Huang et al., 2020). While extensive validation is necessary before the creation of functional organs derived from decellularized ECM scaffolds is feasible, these studies do support the notion that ECMs derived from native tissues produce more physiologic BC populations and airway epithelia in an *in vitro* setting.

### Disease Pathogenesis

It is becoming increasingly apparent that changes in the ECM are associated with the development of many chronic lung diseases. As such, increasing our understanding of the role ECM alterations play in disease pathogenesis offers a new potential route for the development therapies for chronic conditions. COPD, IPF, asthma, and lung cancers are several diseases in which significant changes in the pulmonary ECM have been observed (Fukuda et al., 1989; Bensadoun et al., 1996; Black et al., 2008; Merrilees et al., 2008; Löfdahl et al., 2011; Estany et al., 2014; Eurlings et al., 2014; Abdillahi et al., 2015; Burgess et al., 2016; Burgstaller et al., 2017; Elowsson Rendin et al., 2019). There are very few viable treatments available for these diseases and, in their most serious forms, complete lung transplantation is often the only option (Arcasoy and Kotloff, 1999). Unfortunately, there is a significant lack of suitable donors and, at most, a lung transplantation is a temporary cure with post-transplant survival averaging only 5 years (Arcasoy and Kotloff, 1999; Cypel et al., 2015). Regenerative therapeutic options include: 1) decellularization and repopulation of donor lung and/or animal sourced organs with patient specific stem cells (Cortiella et al., 2010; Badylak et al., 2011b; Au - Bonvillain et al., 2013) (Figure 1B), 2) engraftment of *ex vivo* expanded autologous BCs (Ghosh et al., 2016; Ma et al., 2018) (Figure 1C), 3) derivation of autologous iPSC-derived airway cells (Figure 1C) (Firth et al., 2014; Huang et al., 2014; Miller et al., 2018; Hawkins et al., 2020; Kanagaki et al., 2020; Yamamoto et al., 2020) or 4) activation of endogenous basal stem cells to repair the damaged tissues. We refer the readers to Ikonomou *et al.* who have thoroughly reviewed the current state of cell-based therapies for respiratory diseases (Ikonomou et al., 2019). All of these options rely upon a receptive cellular microenvironment to preserve BC stemness and the ECM is likely a critical component for this. Our current knowledge of the ECM changes linked to asthma, COPD and IPF will be reviewed primarily focusing on changes in the proximal airways.

#### Asthma

Asthma is a chronic inflammatory disease of the airways marked by hyperresponsiveness of the airways to triggers or irritants such as allergens, viruses, and exercise resulting in exaggerated narrowing of the airways (Mims, 2015; Quirt et al., 2018). This causes the individual to experience recurrent bouts of wheezing, breathlessness, feeling tightness in the chest, and/or coughing varying in intensity from mild to, in some cases, fatal severity (Mims, 2015; Quirt et al., 2018). These symptoms are the result of wide-spread airflow obstruction within the lungs. Despite extensive evaluation of changes in airway smooth muscle (ASM) phenotype and ECM interactions there is relatively little known about how ECM changes in asthma regulate epithelial-mesenchymal communication and subsequently BC function. We will summarize some of the key findings specific to the regulation of airway BCs and ECM in asthmatic airways.

It is clear that the profile of ECM proteins in altered in asthmatic airways with the degree of ECM remodeling correlating to the severity of the patient’s symptoms. The deposition of fibronectin, tenascin, hyaluronan, versican, laminin α2/β2, and collagens I, III, and V has been shown to increase, while the deposition of collagen IV and elastin is decreased (Bousquet et al., 1992; Chetta et al., 1997; Roberts and Burke, 1998; Araujo et al., 2008; Nihlberg et al., 2010; Weitoft et al., 2014). When investigating asthma, it is critical to consider complex interactions within the immediate microenvironment of the airway epithelium, a structural and immunological barrier known as the epithelial mesenchymal trophic unit (EMTU) (Vignola et al., 2000; Heijink et al., 2014). Furthermore, the change in deposition of ECM can have considerable changes on matrix stiffness which, in turn, can differentiate fibroblasts to myofibroblasts leading to further matrix production (Shi et al., 2013). *In vitro* experiments replicate this phenotype with TGF-β-stimulated fibroblasts grown on polydimethylsiloxane (PDMS) substrates having increased expression of collagen 1 and alpha-smooth muscle actin indicative of their differentiation to myofibroblasts. As myofibroblast differentiation increased, substrate stiffness also increased from 1 to 50 kPa (Shi et al., 2013). Cells involved in the inflammatory response underlying asthma can interact with, and induce, structural changes in the airway, including synthesis of ECM by fibroblasts (Chan et al., 2006; Shi et al., 2013; Osei et al., 2020). Interleukin signaling has been shown to be one of the key pathways that can stimulate changes in the secretion of ECM proteins including fibronectin and collagen (Chan et al., 2006; Osei et al., 2020). IL-1 expression is increased in asthmatic HBECs compared to those cultured from non-asthmatic lungs, this has a significant impact of pro-inflammatory responses in airway fibroblasts, production of collagen, fibronectin and periostin and leads to abnormal collagen remodeling of the asthmatic-EMTU. Further studies need to be carried out to fully evaluate IL-1 signaling as a therapeutic option for treating chronic asthma (Osei et al., 2020). Another potential target is fibulin-1 (Fbln1) which has recently been shown to both stabilize collagen and other ECM proteins and to regulate the expression of mucins including MUC5AC, and inflammatory mediators, such as CXCL1, in airway epithelial cells (Liu et al., 2017). Another example of changes in asthmatic HBEC regulating the ECM is highlighted in work studying the production of Hyaluronan (HA) a non-sulfate glycosaminoglycan synthesized at the cell membrane. Studies have shown that HA is increased in both bronchioalveolar fluid (Bousquet et al., 1991; Zhu et al., 2010; Ge et al., 2018) and in ECM produced when HBECs of asthmatics are cultured with human lung fibroblasts (Reeves et al., 2018). Its ability to suppress airway inflammation and mucus secretion, indicating that HA may be a potential therapeutic option for reducing remodeling in asthma (Ko et al., 2020).

Other mechanisms driving abnormal ECM composition include increased synthesis of new ECM proteins, decreased activity of ECM-degrading enzymes [metalloproteinases (MMPs) in particular] (Bajbouj et al., 2021), and upregulation of the tissue-specific inhibitors of metalloproteinases (TIMP) (Bajbouj et al., 2021). The role of MMPs in asthma pathogenesis is not as simple as it may seem with disagreement in the field on the destructive vs protective impact of MMPs in chronic lung diseases like asthma (Grzela et al., 2016). Discrepancies may be attributed to the severity of the disease or localization of specific MMP responses (Araujo et al., 2008), significant research still needs to be carried out in this area and likely requires the use of more complex, human tissue-level studies comprising of all the cells in an EMTU. MMP-9 and MMP-12 are increased in the airway smooth muscle of the large airways in fatal cases of asthma (Araujo et al., 2008) and it has been shown that HA may play a role in suppressing MMP9 production and be of potential therapeutic benefit (Ko et al., 2020). More recently a role for MMP2, expressed by HBECs, has been considered in ECM remodeling and data suggests that MMP2 may be key in mediating crosstalk between HBEC and their underlying myofibroblasts in the EMTU and that a net change in MMP2 activity in asthma may promote fibrosis and increased deposition of ECM, despite its role in ECM degradation (Xu et al., 2002). This contrasts data from mice where MMP2 is proposed to provide a protective role (Takahashi et al., 2019). Most recently a study of over 80 bronchial biopsies identified significant overexpression of MMP-10 and MET in biopsies with high mucosal eosinophil numbers. These genes are involved in ECM organization and their increased expression in these tissues corelated to increased submucosal thickness (Kuo et al., 2019). Mechanisms pertaining to MMP regulation and function in asthma remain to be fully validated and regulation of MMP production is likely to be a potential therapeutic option in the treatment of chronic asthma (Kuo et al., 2019).

The data in *in vitro* models of asthma are supported by changes observed in the lungs of asthmatic patients. Recently, Vieira Braga et al. completed a comprehensive scRNAseq profile of biopsies from asthmatic airways. The data revealed a significant increase in goblet cell numbers compared healthy control samples supporting prior observations in asthmatic lung tissues (Vieira Braga et al., 2019). The transcriptome of the goblet cells was deviant from goblet cells from control lungs with an up regulation of proinflammatory and remodeling genes including nitric oxide synthase 2 (NOS2), Carcinoembryonic antigen-related cell adhesion molecule 5 (CEACAM5), and Cystatin-SN (CST)1. Furthermore, asthmatic airways present a novel cell state the authors label as mucous ciliated cells which resemble ciliated cells (FOXJ1 expressing), but also co-express some mucous genes including MUC5AC and CEACAM5 (Vieira Braga et al., 2019). In addition, these mucous ciliated cells express the genes CST1 and Periostin (POSTN) that have been previously shown to play a role in the remodeling of asthmatic airways. Clinical trials to evaluate allergen exposure in asthmatic patients have also shown that exposures correlate with significant transcriptomic changes in the airway epithelium, including upregulation of markers of cell proliferation, and differentiation, as well as extracellular proteins, interleukin receptors, and transcription factors in subjects clinically diagnosed with asthma (Lilly et al., 2005). These genetic changes suggest that the epithelial response to allergens involves genes likely to change the function of airway epithelial cells. Despite detailed analysis of changes in the extracellular matrix and cellular phenotype being studied in asthma there is still a paucity of data to understand how the changes in ECM influence the changes in BC phenotype and differentiation that have been observed.

Inhaled corticosteroids and beta-2-agonists are commonly used as asthma treatments to reduce inflammation and relax the airway smooth muscle; these treatment options are effective at preventing exacerbations, but they are not able to treat airway remodeling (Castillo et al., 2017). More recently, therapy routes are being explored that seek to target the ECM and airway smooth muscle as a means to reverse asthmatic airway remodeling (ClinicalTrials.gov Identifier: NCT03388359). While still in early development, this seems to be the most promising avenue for restoring asthmatic airways and potentially eliminating completely the associated symptoms.

#### Chronic Obstructive Pulmonary Disease

COPD is a progressive disease that is characterized by emphysema, reduction of gas exchange within the lung, chronic bronchitis, constant irritation, inflammation, and excess mucus production in the airways. Cigarette smoking is the primary cause of COPD with around 75% of the patients being current or past smokers. COPD is currently the 4th leading cause of death in the United States, and it is predicted that it will soon be the 3rd. The ECM of COPD patients shows abnormalities in both structure and composition. Finely disrupted fibers, electron-dense deposits and vacuoles, and indications of abnormal elastogenesis are seen in the elastic fibers (Fukuda et al., 1989). Further, the overall presence of elastic fibers is reduced in the small airways, possibly contributing to the development of airflow obstruction in the alveoli (Black et al., 2008; Merrilees et al., 2008; Eurlings et al., 2014). In contrast, other ECM elements, including tenascin-C (Löfdahl et al., 2011; Annoni et al., 2012), fibronectin (Annoni et al., 2012), versican (Merrilees et al., 2008), and collagens (Eurlings et al., 2014; Abdillahi et al., 2015) have been shown to be overexpressed in COPD. It is suspected that the changes in the ECM contribute to the dysfunction of the airway epithelium observed in COPD by regulating the function of airway BCs, possibly by acting as storage for inflammatory mediators, growth factors and cytokines. Basal stem cell number, self-renewal, and multipotency are all reduced in COPD, with impaired lung function as a consequence of impaired BC differentiation (Ghosh et al., 2017). Instead of a multipotent basal stem cell a more differentiated progenitor cell progeny persists. Squamous metaplasia is a hall mark of COPD lungs in which an increase in proliferative TP63 expressing basal cells contributes to significant airway thickening (Araya et al., 2007). Upregulation of squamous cell markers including IVL and KRT6 is observed in the absence of mucociliary differentiation and correlates to the severity of COPD (Araya et al., 2007). Several studies have suggested that a partial EMT transition is part of this process and characteristic of COPD occurring through TGF-β1-dependent regulation of BCs (Gohy et al., 2015). In these instances, the EMT transition is characterized by upregulation of EMT associated markers Twist1, Twist2, Snai2 vimentin and N-cadherin and a reduction in the expression of E-cadherin (Nishioka et al., 2015). In *in vitro* assays, however, the abnormal mesenchymal phenotype only persists for two weeks in ALI models, after which mesenchymal markers progressively declined, suggesting that there are other *in vivo* microenvironmental factors necessary to maintain EMT or that EMT is specific to *in vitro* culture systems which mimic airway epithelial repair (Gohy et al., 2015). In this particular study there was a significant difference in EMT between control and COPD patient derived basal cells suggestive of an augmented EMT phenotype in COPD and this is supported by evidence of active EMT in patient tissues (Sohal et al., 2011). EMT has previously been described as an active process in the large and small airways of patients with mild to moderate COPD validating these observations (Sohal et al., 2010; Soltani et al., 2010; Soltani et al., 2012; Milara et al., 2013; Nishioka et al., 2015). Most recently, Cullin4A (CUL4A), an E3 ubiquitin ligase associated with EMT in non-small cell lung cancer, is also overexpressed in the lung epithelium of COPD patients and could be associated with the poor differentiation and function of the small airway epithelium (Ren et al., 2019) There still remains some controversy over the specific role for an active EMT transition in the pathogenesis of COPD. What can be concluded is that a significant change in the matrix associated genes and a reprogramming of airway BCs are core factors in COPD pathophysiology, however significantly more work needs to be completed to fully understand the mechanisms driving this BC dysfunction.

A role for TGF-β1 impairing mucociliary differentiation of ALI cultures, offers a potential explanation for the reduced number of ciliated cells observed in COPD patient airway samples (Gohy et al., 2019). Interestingly, HBECs grown on decellularized bronchial scaffolds from COPD patients show patterns of gene expression during early differentiation that deviate from cultures on healthy scaffolds (Hedström et al., 2018). These differences indicate altered activity of upstream mediators of regeneration and remodeling through hepatocyte growth factor (HGF) and TGF-β1 respectively (Hedström et al., 2018). HGF activity was down regulated which impairs proliferation and survival of airway epithelial cells, while several genes known to be induced by TGF-β1 were upregulated indicating increased TGF-β1 activity and consequently ECM dysregulation (Hedström et al., 2018). While the precise mechanisms have yet to be unraveled, these results indicate that the ECM changes that accompany or prelude COPD are involved in the further progression of the disease. A decrease in ciliated cell differentiation is likely congruent with squamous BC metaplasia that is also associated with COPD as a result of smoking and is thought to be induced, at least in part, through chronic inflammation (Herfs et al., 2012). Compared to healthy pseudostratified epithelium from non-smoking patients, squamous metaplasia and epithelial hyperplasia are accompanied by a significant increase in proinflammatory cytokines TNF-α, IL-1β, and IL-6 (Herfs et al., 2012). Changes in signaling mediated by extracellular vesicles from (Herfs et al., 2012; Gohy et al., 2015; Brandenberger and Mühlfeld, 2017) HBECs are able to induce fibroblast differentiation to myofibroblasts in *in vitro* experiments (Xu et al., 2018). It will be interesting to further study these effects in *in vivo* models (Fujita et al., 2015; Xu et al., 2018). Such increases in the myofibroblast population in the lungs may in part explain the elevated levels of some ECM components observed in COPD. The study by Xu and colleagues also demonstrates how changes induced in one cell type, in this case HBECs, can significantly impact other cell types (Ghosh et al., 2017). Refining our understanding of the relationship between ECM changes and abnormal BC phenotype in COPD can open new possibilities for the early identification and treatment of the disease.

#### Idiopathic Pulmonary Fibrosis

IPF is another chronic lung disease in which significant modifications of the ECM and changes in BC phenotype have been observed. IPF is characterized by significant deposition of ECM components throughout the alveolar parenchyma and, to a lesser extent, the terminal airways (Burgess et al., 2016). This process is facilitated by fibroblastic foci, which are clusters of myofibroblasts and fibroblasts, neighboring apoptotic or hyperplastic alveolar epithelial cells (Burgess et al., 2016; Burgstaller et al., 2017). Abnormal populations of both fibroblasts and myofibroblasts are present in the foci. Fibroblasts exhibit increased expression of hyaluronan synthase 1 (HAS1), hyaluronan synthase 2 (HAS2), fibrillin 1 (FBN1), and chemokine ligand 14 (CXCL14), and myofibroblasts fibrillar collagens and smooth muscle alpha-2 actin (ACTA2) (Adams et al., 2020). An overall decrease in the proportion of alveolar epithelial cells is reported as well (Adams et al., 2020). Repeated lung injury and repair are responsible for ongoing destruction of the elastic parenchymal lung tissue which is then replaced by scar tissue that develops into fibroblastic foci (Burgstaller et al., 2017). As the lung tissue is progressively remodeled, bronchiectasis, thicken interlobular septae, and subpleural honeycombing develop (Burgstaller et al., 2017).

Similar to the response to acute lung injury, the development of fibrosis in IPF seems to result from exudate within the airspaces, and fibroblasts increase collagen and fibronectin synthesis throughout the process (Kuhn et al., 1989; Elowsson Rendin et al., 2019). The ECM compounds versican and tenascin C have also been shown to be upregulated in the fibroblastic foci (Bensadoun et al., 1996; Estany et al., 2014). However, the basement membrane portion of the ECM is depleted of proteins such as laminin and collagen IV and shows overall disorganization (Elowsson Rendin et al., 2019). Increases in the elastic fiber proportion of the ECM associated with IPF have been correlated to worse patient survival outcomes because excess elastin alters the stiffness of the lungs which increases the effort of breathing due to higher elastic recoil (Enomoto et al., 2013). IPF lungs show a 60% increase in stiffness and three-fold increase in tissue density compared to healthy lungs (Elowsson Rendin et al., 2019).

The ECM changes originating from the fibrotic foci seem to have a significant effect on the overlying basal epithelium. The airway epithelium over the fibrotic foci contains a layer of TP63-positive BCs and lacks ciliated and goblet cells (Jonsdottir et al., 2015; Adams et al., 2020). Increased expression of KRT14, Vimentin, and N-cadherin is also observed in the epithelial cells; however, the underlying foci shows N-cadherin and E-cadherin-positive cells (Jonsdottir et al., 2015). These alterations indicate that BCs acquire mesenchymal traits due to signals originating from the foci or, alternatively, that the EMT of the epithelium directly contributes to foci formation (Jonsdottir et al., 2015). Distally, IPF lungs demonstrate a larger population of KRT5 positive cells, a subpopulation of KRT5/KRT14 positive cells, and morphological changes in these BCs (Smirnova et al., 2016). In this study, KRT5/KRT14/TP63 positive BCs were totally absent in the healthy distal lungs, while they were abundantly found in the IPF lungs (Smirnova et al., 2016). The KRT14 positive population also did not express bronchial or alveolar differentiation markers in IPF (Smirnova et al., 2016). In a recent publication, Adams *et at.* demonstrated a more precise characterization of the abnormal BC phenotypes observed in IPF lungs (Adams et al., 2020). They report a population of BCs, aberrant BCs, unique to IPF that express BC markers TP63, cytokeratin 17 (KRT17), laminin subunit beta-3 (LAMB3), and laminin subunit gamma-2 (LAMC2), but also lack other accepted BC markers such as KRT5 and KRT15 (Adams et al., 2020). These aberrant BCs also show evidence of EMT, senescence-related genes, and high expression of IPF-associated molecules including matrix metallopeptidase 7 (MMP7) (Adams et al., 2020). Aberrant BCs are found consistently in the epithelial layer covering fibroblastic foci (Adams et al., 2020). The exact mechanisms of IPF development related to abnormal ECM production and BC phenotype remain unknown, however the significant upregulation of ECM-related (such as FN1, COL1A1 and VCAN) and ECM-interacting genes (such as Integrins B8, AV and B6) in the aberrant basaloid cells suggests that changes in the ECM may influence the change in cellular phenotype. The publication of large scRNA sequencing studies comparing IPF basal cells to non-IPF basal cells may start to provide clues regarding the phenotypic switch in basal cell phenotypes; however further studies will be essential to uncover the relationship between ECM changes and BC phonotype if effective therapies are to be developed (Carraro et al., 2020b).

### Conclusion and Future Implications

An absolute understanding of the pulmonary ECM and its interactions with, and effects on, the cells of the lung may be the key to developing everything from a physiological airway model to a functional recellularized lung. The ECM is part of the complex environment of the lung, with regional variations, that supports and maintains the overlying tissue. As BCs are the primary stem cells of the lung and are positioned to have the most direct contact with the ECM, deciphering their reliance on the ECM for the maintenance stemness and differentiation capacity will allow for advancements in airway modeling and understanding of disease pathogenesis. Regional variations in BC distribution, function and ECM localization to specific BC niches are still relatively poorly understood. Furthermore, we need a greater understanding of how age-related changes in ECM correlate to changes in basal cell function as we progress toward cellular therapy and regenerative therapeutics for the lung. Continued research in the areas discussed in this review will have momentous implications for the modeling of healthy and diseased airways, understanding the relationship between ECM alterations and abnormal BCs in chronic lung disease, therapy development for such diseases, and decellularization/recellularization capacity.

The current standard methods of BC culture and airway modeling fall short in producing cells and tissue that fully maintain their native phenotype. While commercial culture ECMs, medias, and differentiation platforms, such as Matrigel®, BEGM with Y-27632, and ALI inserts respectively, have improved the culture of airway cells considerably, abnormal gene expression and differentiation is still observed. If our airway models do not entirely simulate *in vivo* cells and conditions, there are caveats associated with experiments conducted on them. By improving our understanding of the *in vivo* pulmonary ECM and how it supports BCs and other cell types, we will become better equipped to create an *ex vivo* culture ECM that supports native conditions. Variables such as stiffness, stretch, and molecular composition are proving to be of particular importance. A promising direction of ECM research is the conversion of the 3-D native lung ECM scaffold into a hydrogel that can be plated on a 2-D surface. Ideally, this will allow for the preservation of the ECM components and stiffness found *in vivo*. This technique also offers the option of using ECM from diseased lungs to study disease-specific ECM alterations and the subsequent changes in cellular response. It will be interesting to follow this, and the progression in 3-D bioinks where the impact of 3-D structure and curvature of the airways can also be accounted for in the regulation of BC function. Complete understanding of BC-ECM interactions may have a significant impact on our capacity to efficiently repopulate decellularized lungs/tracheas to generate functional tissues for engraftment. While there has been substantial progress made on techniques for decellularization and recellularization there is still a paucity in the characterization of the ECM, adherence and functional quality of the recellularized tissues [reviewed in (Uriarte et al., 2018)]. Current protocols for decellularization vary considerably in reagents used and length of digestion which pose challenges in balancing the structural and chemical composition of the ECM with complete cellular removal. Evaluation of the remaining ECM including the level of residual proteins will require more quantitative proteomic approaches (Schiller et al., 2015; Gilpin et al., 2017), in addition to a more complete understanding of basal stem cell homing, niche, subtypes and differentiation will be critical as the field of lung tissue engineering moves forward.

Abnormal ECM and BC phenotype appear to be closely related in the context of chronic lung disease. As such, BCs have become a target for regenerative therapy development in conditions such as COPD and IPF where lung transplantation is currently the only curative option. Engraftment of *ex vivo* expanded autologous BCs, derivation of autologous iPSC-derived BCs, and activation of endogenous basal stem cells to repair damaged tissues are currently under investigation as patient-specific cell therapies that could provide a superior alternative to transplantation. However, these techniques rely upon a receptive cellular microenvironment for both *ex vivo* expansion and *in vivo* engraftment, further highlighting the necessity for improved knowledge of ECM mechanisms in the context of native physiology and culture platforms.

Perhaps the most impressive and challenging application of ECM-BC research is the potential to repopulate a decellularized ECM scaffold to create a functional lung. Decellularization of donor lungs and recellularization with autologous stem cells is thought to be a solution to overcome the deficiency of donor lungs suitable for direct transplantation and avoid complications such as organ rejection. While there has been some success with simpler structures such as the trachea alone, researchers still face significant challenges in the complex and diverse structural and biochemical environment of the whole lung. Such challenges include the effective removal of cellular material while preserving the structural and mechanical properties of the ECM, seeding of cells to maintain compartmentalization (i.e., airways vs. vasculature), and differentiation to produce functional gas exchange units. Advancements in our awareness of the often subtle, differences in ECM composition in different regions of the lung and how these differences produce diverse tissue types will aid in our ability to contain seeded cells to their designated area and direct them to differentiate appropriately.

While at the surface level the ECM may appear to be simple scaffold, in reality it plays a critical role in lung physiology through mechanical and biochemical factors. Further study of the pulmonary ECM and its relationship with cells will progress airway modeling, therapy development for chronic diseases, and overall understanding of lung physiology.
